# Nutritional Quality of Dry Vegetable Soups

**DOI:** 10.3390/nu11061270

**Published:** 2019-06-04

**Authors:** Leo van Buren, Christian H. Grün, Silke Basendowski, Martin Spraul, Rachel Newson, Ans Eilander

**Affiliations:** 1 Unilever R&D Vlaardingen, Olivier van Noortlaan 120, 3133 AT Vlaardingen, The Netherlands; leo-van.buren@unilever.com (L.v.B.); christian.grun@unilever.com (C.H.G.); newson_rachel@lilly.com (R.N.); 2 Unilever R&D Heilbronn, Knorrstrasse 1, D-74074 Heilbronn, Germany; silke.basendowski@gmx.de (S.B.); martin.spraul@unilever.com (M.S.)

**Keywords:** dry soups, dry vegetables, nutritional composition, nutritional density score

## Abstract

Dry soups with vegetables are often perceived as having low nutritional quality, but there are only limited data on the nutritional value of dry soups. Therefore, we measured the nutritional composition of dry vegetable powders used in dry soups and compared the results with published data on fresh and cooked vegetables. We also analyzed the nutritional composition of dry vegetable soups and compared these with published data on home-made and other soups. Dietary fiber, minerals, vitamins, and carotenoids in dry vegetables powders and soups were analyzed. Based on these data, a nutrient density score was calculated as measure of overall nutritional quality. Nutrient density scores for fresh and cooked vegetables, as well as home-made and other soups, were calculated based on the United Stated Department of Agriculture (USDA) and “Bundeslebensmittelschlüssel” (BLS) food composition data. The nutrient density scores of dry vegetable powders did not systematically differ from cooked vegetables. Nutrient contributions to European Food and Safety Authority (EFSA) dietary reference intakes per 250 mL serving of soup ranged from 11–45% for fiber; 3–23% for iron, magnesium, and zinc; 8–22% for potassium; 11–15% for vitamin A; 2–17% for B-vitamins; and 2–15% for vitamin K. The nutrient density scores of dry vegetable soups were in the same order of those of home-made and other soups. These data indicate that dry vegetable soups, like home-made soups, can deliver a significant part of recommended daily nutrient and vegetable intake.

## 1. Introduction

Multiple studies have indicated that a higher intake of vegetables is associated with a reduction of cardiovascular diseases (CVDs) and possibly with a reduction of obesity, type 2 diabetes, chronic respiratory diseases, and some types of cancer [1,2,3,4,5,6]. The benefits of vegetables on the prevention of non-communicable diseases (NCDs) may be explained by their relatively high content of micronutrients, antioxidant compounds, polyphenols, and fibers, which may each counteract the biochemical processes that cause CVDs and other NCDs [7,8]. According to the Global Dietary Database 1990–2010, vegetable (including legumes) intake ranged from 35–493 g/day, with a mean intake of 209 g/day. The authors concluded that the intake was, in most countries, lower than recommended to prevent chronic diseases [9].

Health authorities such as the World Health Organization/Food and Agriculture Organization (WHO/FAO) recommend a daily intake of ≥400 g/day fruits and vegetables, which corresponds to five or more servings of 80 g/day [10]. Many countries add to this recommendation that three out of these five servings should come from vegetables (≥240 g/day). WHO/FAO does not include tubers such as potatoes and cassava in their definition of vegetables [11]. However, unclarity exists as to whether all vegetable sources (e.g., sauces, meals, and soups) can be regarded as a vegetable. In the UK, for example, processed vegetables in frozen, canned, and dried form, as well as vegetable juices, are included to meet the recommendation [12]. At the same time, dried vegetable powders do not qualify to count as a vegetable portion according to guidelines on calculating and communicating fruit and vegetable portions in composite foods by the Institute of Grocery Distribution in the UK [13]. As a consequence, most definitions do not include vegetables in dried soups, although these products may be a significant source. A general lack of adequate data on the nutritional composition of dried vegetables and dried vegetable soups may underlie these decisions.

The drying or dehydration of vegetables is applied to preserve vegetables and their flavors and nutrients. Drying methodologies include osmotic, convective, fluidized bed, ohmic, microwave, vacuum, and freeze-drying techniques [14]. Before the actual drying phase, there is a pre-processing step to remove foreign materials and to select, wash, occasionally peel, deseed, and reduce the size of the vegetables. Techniques such as blanching, acid treatment, and the application of coatings are regularly applied to preserve the quality of the vegetables [14]. Though the industrialized drying process is optimized to maximally preserve food quality, both pre-processing and drying may degrade certain nutrients, particularly heat-labile nutrients, such as flavonoids, carotenoids, and vitamins [15]. As chemical analyses of nutrients are expensive, there are limited published data on the nutritional composition of dried vegetable soups. Data on these products in food composition tables (FCTs) originate in most cases from a limited number of data sources that may be based on outdated analytical methods. To judge whether the vegetables in these soups qualify as vegetables for recommended intakes, there is a need for better data on the nutritional composition of dried vegetable soups.

The current study aimed to measure the nutritional composition of dry vegetable powders used in dry soups and to compare these with published data of fresh and cooked vegetables. Its second aim was to assess the nutritional composition of dry vegetable soups and to compare these with published data of home-made and other soups. Based on these data, the fresh vegetable equivalents of dry vegetable powders in dry soups will be determined.

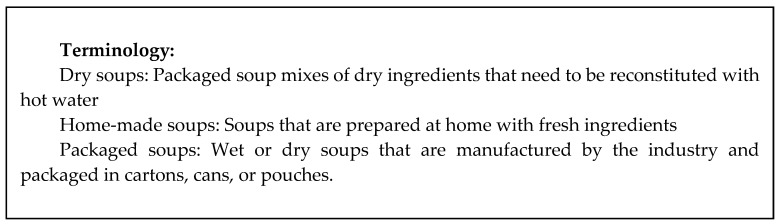



## 2. Materials and Methods

### 2.1. Nutrient Content Analyses of Dry Vegetable Powders and Dry Vegetable Soups

We analyzed the nutrient content of dry vegetable soups and also analyzed the dry vegetable powders that were used as main ingredients for the soups. Therefore, four commonly consumed vegetable soup varieties commercially available on the European market, including dry tomato, onion, pumpkin, and legumes/pulses (lentil and bean) soups, were selected for the nutrient analyses. A fifth soup type comprising a mixture of vegetables was added to cover a wider range of variety in vegetable ingredients such as celeriac, carrot, leek, beetroot, and broccoli. The trade names and composition of all soups can be found in the Supplemental data S1. For each type of soup, two varieties were selected so that in total 10 dried soup products were analyzed.

Three packs of each soup product were obtained directly from the filling line in the different Knorr factories in Europe. We also took three samples of 200 g of the dry vegetable powder that was the main ingredient of each soup variety (covering 39–96% of the ingredients), including tomato, onion, pumpkin, and lentil powder.

Samples were shipped at ambient temperature to the laboratory “Institut Kuhlmann GmbH Analytik-Zentrum” (Ludwigshafen, Germany), where samples were homogenized and subsequently analyzed in triplicate. Insoluble and soluble high and low molecular weight fiber were determined by AOAC 2009.01 and expressed as total dietary fiber. Magnesium, iron, zinc, potassium, and sodium were determined via microwave digestion followed by analysis by ICP-OES (ISO-16943). β-carotene, lycopene, and lutein were determined by HPLC Diode-Array Detection (HPLC-DAD). Provitamin A β-carotene was converted to retinol activity equivalents using a factor 12. Thiamin (vitamin B1) and riboflavin (vitamin B2) were determined by HPLC with fluorescence detection; niacin (vitamin B3), pantothenic acid (vitamin B5) and pyridoxine and pyridoxal (vitamin B6) were determined by HPLC-MRM-MS, and total folate was determined by measuring the turbidity of *Lactobacillus casei* growth according to AOAC 2004.05. Total vitamin C was determined by extracting the samples with meta-phosphoric acid solution followed by a homocysteine treatment and quantification by HPLC-DAD. Phytomenadione (vitamin K1) was determined after fat reduction with lipase and extraction with hexane, and it was quantified by HPLC with fluorescence detection.

Because the amount of dry soup to prepare a serving of 250 mL ranged from 16 g dried powder for onion soup to 67 g for the dried lentil soup, the nutrient values of the dried soup powders were expressed per serving size to allow a practical comparison between soup varieties.

The nutrient values of the dried powders and soups per serving were compared to the dietary reference intake (DRI) values by the European Food Safety Authority [16].

### 2.2. Nutrient Density Score Calculation to Compare Nutritional Quality of Dry Vegetable Powders and Soups with Published Data of Vegetables and Soups

A nutrient density score was calculated as measure of overall nutritional quality of each dried vegetable powder and soup, based on a method by Drenowski et al. [17]. For each individual nutrient in the vegetable powder or soup, a nutrient density score was calculated by dividing the nutrient content per 100 g product by the European Food and Safety Authority (EFSA) dietary reference values, which was then divided by the energy density and subsequently multiplied by 100. The overall nutrient density score for each product was calculated as the sum of all individual nutrient density scores of beneficial nutrients (i.e., total dietary fiber, magnesium, iron, zinc, potassium, vitamin A, thiamin, riboflavin, niacin, pantothenic acid, pyridoxine, vitamin C, and vitamin K1) minus the sum of the nutrient density scores of negative nutrients (i.e., sodium). The energy content of the dried powders and soups was not analyzed and therefore derived from the food composition table values for cooked vegetables (for powders) and nutritional information table on pack (for soups).

To allow for comparison of the nutrient density scores based on the analyzed data of the dry vegetable powders with the respective fresh and cooked vegetables of the same variety (i.e., tomato, onion, pumpkin, lentil, and mixed vegetables), the overall nutrient density scores of the latter were calculated based on published data from the United Stated Department of Agriculture (USDA) National Nutrient Database [18] and the German “Bundeslebensmittelschlüssel” (BLS) version 3.01 [19]. To allow for a comparison of the five varieties of dry vegetable soups (i.e., tomato, onion, pumpkin, lentil, and mixed vegetables) with respective home-made and other soups, overall nutrient density scores were calculated for home-made and other soups with similar main vegetable ingredients. Data on vegetables from mixed dishes, soup-based sauces, and stews were not included. Soup was classified as home-made when this was included in the description of the product in the database. Types of soups other than dry and home-made such as packaged soups (e.g., canned) and soups consumed out of home were classified as other soups.

Some nutrients were not included in the overall nutrient density score of particular vegetable powder and soup varieties because these were not analyzed or were below the detection level. This was the case for vitamin C in all except tomato soups, vitamin K1 in pumpkin soups, and vitamin A in onion, pulses, and legume soups.

### 2.3. Calculation of Fresh Vegetable Equivalents in Dry Vegetable Soups for Comparison with Vegetable Recommendation

To assess the vegetable content of the dry vegetable soups, we calculated fresh vegetable equivalents by multiplying the quantity of each dry vegetable powder in the soup with a factor to account for rehydration. This factor was obtained by dividing the weight of the dried vegetable powder (corrected for remaining water content as provided by the ingredient suppliers) with the dry weight of the fresh vegetable. Fresh vegetable equivalents of the dry soups were compared to the vegetable intake recommendations of 240 g/day from the WHO/FAO [10].

## 3. Results

### 3.1. Nutrient Content of Dry Vegetable Powders

Table 1 provides the analyzed nutrient content of the dry vegetable powders used as main ingredient of the dry vegetable soups. Onion powder was more than three times higher in dietary fiber (61.5 g/100 g) than tomato, pumpkin, and lentil powder (17.0–17.9 g/100 g). Lentil powder contained two times more iron (7.8 mg/100 g) and zinc (3.8 mg/100 g) than the other vegetable powders (3.3–4.8 and 1.3–1.7 mg/100 g, respectively). Tomato powder contained more niacin (11.9 mg/100 g) than the other vegetable powders (1.0–4.0 mg/100 g), and it also contained significant amounts of provitamin A from β carotene (210 µg RAE/100 g), vitamin C (151 mg/100 g), and vitamin K1 (25.5 µg/100 g).

### 3.2. Comparison of Nutrient Density Scores of Dry Vegetable Powders with Fresh and Cooked Vegetables

Table 2 shows the overall nutrient density scores of the analyzed dry vegetable powders next to the nutrient scores of the respective fresh and cooked vegetables according to nutrient content in the food composition databases. The overall nutrient density scores ranged from 142 for onion powder to 394 for lentil powder. For onion, pumpkin, and lentil, the overall nutrient density scores did not differ between dry and cooked vegetables. The dry tomato powder (315) had a lower nutrient density score than cooked (425) and fresh (473) tomatoes.

### 3.3. Nutrient Content of Dry Vegetable Soups

Table 3 shows the nutritional composition of the dry vegetable soups per serving of 250 mL. The lentil soup contained the most nutrients per serving, with contributions to the DRI per serving of 45% for fiber, 17–23% for minerals, 8–17% for B-vitamins, and 15% for vitamin K. For the other soups, nutrient contributions to the DRI per serving were 11–21% for fiber, 3–6% for iron, magnesium and zinc, 8–22% for potassium, 11–15% for vitamin A, 2–12% for B-vitamins, and 2–14% for vitamin K. Vitamin C could only be detected in the tomato soup, where one serving contributed to 18% DRI. Folic acid could not be detected in any of the soups.

### 3.4. Comparison of Nutrient Density Scores of Dry Vegetable Soups with Home-Made and Other Soups

**Figure 1:**
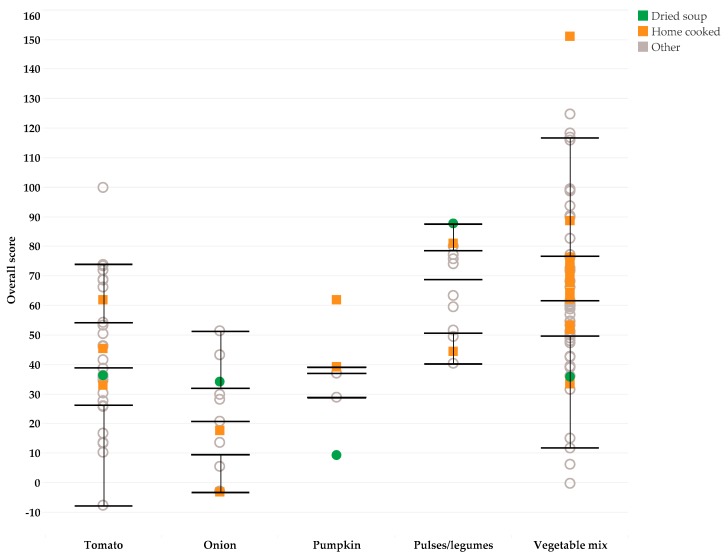
Nutrient density scores of dry vegetable soups with home-made and other soups.

A total of 125 soups in the USDA and BLS food composition tables met the selection criteria for comparison with dry vegetable soups; 22 were classified as home-made soups, and 102 were classified as other soups. The nutrient density score of these soups ranged from 77 to 151. Figure 1 shows that there were no systematic differences between the nutrient density scores of the dried soups and that of other types of soup. As compared to home-made soups, the nutrient density scores of the dry soups were relatively low for pumpkin soup and relatively high for onion and pulses/legume soup.

### 3.5. Fresh Vegetable Equivalent Content of Dry Vegetable Soups

The vegetable powder content of the dry vegetable soups ranged from 9–41 g per serving of 250 mL (Table 4). This is equivalent to 0.9–2.5 portions of fresh vegetables per serving and corresponds with 29–82% of the daily recommended vegetable intake of 240 g/day. The mean fresh vegetable equivalent content of the dried soups was 120 g, which corresponds to 1.5 portion equivalents of vegetables and 50% of the recommended intake.

## 4. Discussion

This study aimed to measure the nutritional composition of dry vegetable powder and soups and to compare the nutrient density scores of dry vegetable powders with that of published data from respective fresh and cooked vegetables and home-made and industrially prepared soups. Our data indicate that the nutritional quality of dry vegetable powders is of similar magnitude as that of cooked vegetables. As a consequence, dried vegetable soups made from these powders can, like other types of vegetable soups, significantly contribute to the recommended daily intake of fibers, minerals, and vitamins.

Our study provides new data on the nutritional composition, including fiber, 13 micronutrients, and sodium of different dried vegetable soups, by using validated methods. We compared these new data against a substantial amount of data from home-made and industrially prepared soups in USDA and BLS food composition tables. A limitation is the explorative design of our study; due to scarce and heterogenous published data, we could not formulate a quantitative hypothesis on the differences between the nutrient composition of dry vegetable powders and soups and that of respective fresh and cooked vegetables and other soups. To confirm our observations, a more comprehensive sampling methodology and statistical approach to address variation in factors that influence the nutritional quality would be needed. Due to practical constraints, we had to use different methodologies to compare the nutrient content and nutritional quality of the different vegetables and soups (i.e., analyzed data for the dry vegetable powders and soups versus published data of fresh and cooked vegetables and other soups). For example, fiber content was considerably higher in our dry vegetable soups than that of respective products in the food composition tables, which may be explained by differences in methodology of analyses and in fiber definitions. Furthermore, we had to discard some of the nutrient content data due to failure of analyses—the unexpected high and highly variable folate levels in the soups due to influence of lipids in the soup matrix on the assay, for instance. A method based on HPLC-DAD-MS for quantifying individual folate homologues that we have used to measure nutrient the retention of folate during soup preparation in a separate study yielded more reliable results (see Supplemental data S2) and may be recommended for the analysis of folate in dry soups in the future.

We used nutrient density scores as a marker for the nutritional quality of the soups and vegetables. It should be noted that these scores cannot be directly related to impact on nutrition and health. For example, the food matrix in which the nutrients are present and form of the product (e.g., fresh, dried, or composite food) could influence the bioaccessibility and bioavailability of the nutrients and, subsequently, impact health [20]. Therefore, the nutrient density scores should rather be used to rank foods within a food category—to compare nutritional quality of different preparation forms (dried, fresh, and cooked) of a specific product or food (in this case vegetable powders and soups), for instance. In the current study, we found that the nutritional quality of the dry vegetable powders was generally similar to that of cooked vegetables, as well as for the fresh equivalent, in the case of onion. In contrast, the nutrient density scores for cooked and fresh tomato were considerably higher than for dry tomato powder due to the relatively high vitamin C level in cooked tomato. When vitamin C was not included in the nutrient density score calculation, scores for dried tomato (255) and for cooked tomato (219–431) were of similar range.

The nutrient content of vegetable crops is determined by many factors, including the type of soil (e.g., pH, water holding capacity, porosity, cation exchange capacity, and mineral composition), climate (e.g., light intensity, temperature, and rainfall), crop variety, management practices (e.g., pesticides and fertilizers), maturity, postharvest handling, and storage [21]. Differences in one or more of these factors may explain part of the variation in the nutritional quality within and between the differently processed vegetables in our study. A comparison of dried, cooked, and fresh vegetables that are grown, harvested, and stored under the same circumstances would be needed to confirm our findings.

Our study shows that dry soups made of lentils and pulses deliver almost twice the amounts of micronutrients as compared to the other dry soups. This was also reflected in the nutrient density scores. Though most of the nutrients that were measured in the dried vegetable soups most likely originate from the dried vegetable powders, we found considerably higher levels for some of the B vitamins than what would be expected from the soups’ vegetable contents. We assume that these higher levels may be attributed to added yeast extract, as this ingredient has been reported to be particularly rich in thiamin, riboflavin, and niacin [22,23,24]. Similarly, the higher potassium levels in tomato and onion soups may be explained by added potassium chloride as a salt-replacing ingredient.

Vitamin C could only be detected in dried tomato soups, which contained 18% DRI. It is known that vitamin C is heat-labile, and major losses are expected during cooking. In order to obtain better insights in nutrient retention during the drying and cooking processes of dried and equivalent home-made soups, we performed an additional study on the retention of a number of key nutrients in tomato, onion, and lentil soups (see Supplementary Material S2 and Figure S1 for methods and results). This retention study showed that 43% of vitamin C levels in dried tomato soups were retained during cooking, similar to home-made tomato soups, in which 36% of vitamin C levels were retained. For lycopene, folate, and potassium (data not shown) there was also no clear differences in nutrient retention between the prepared dried vegetable soups and home-made soups. This is confirmed by our finding that nutrient density scores of the analyzed dried soups had nutrient density scores in the same range as those of home-made and other soups based on data from FCTs.

Our data also shows that the dried vegetable soups can contain between 1–2.5 portion equivalents of vegetables, thereby contributing to 30–80% of the daily vegetable recommendation of 240 g/day. Furthermore, we found that most of the fiber and nutrients from the vegetable ingredients remain present in the dried soups and that the nutrient density of dried soups is similar to that of home-made and other vegetable soups. However, as soups also contribute significantly to sodium intake (36–45% of the daily recommendation in one serving), soup consumption should be kept within limits of dietary guidelines, and efforts should be made to lower the sodium content of soups.

Lastly, health authorities are increasingly shifting their recommendations towards healthy and sustainable diets with an emphasis to consume more plant-based foods, such as fruits, vegetables, whole grains, and pulses [25]. Drying is a process to preserve foods with the potential to increase the plant-based food supply worldwide by preventing post-harvest losses. A recent study showed that, on a per serving basis, dried soups are more environmentally sustainable compared to wet soups, mainly because dried products have a lower mass and therefore require less packaging and are more efficient to transport [26]. While dried products such as dry vegetable soups may be perceived by consumers to be of low nutritional quality, data from the current study indicate that dried vegetable soups can be as nutritious as home-made soups, and, as such, could help to increase vegetable intake and fit in a healthy and sustainable diet.

In conclusion, the nutritional value of dry vegetable soups does not seem different from that of home-made soups. Dry vegetable soups can be considered a suitable source of vegetables and nutrients and may deliver a significant part of recommended daily nutrient and vegetable intake.

## Figures and Tables

**Table 1:** Mean nutrient content of dry vegetable powders.

Nutrient Per 100 g	Tomato	Onion	Pumpkin	Lentil
Total dietary fiber (g)	17 ± 0.2 ^1^	62 ± 2	18 ± 0	18 ± 0
Magnesium (mg)	166 ± 2	112 ± 1	128 ± 2	104 ± 1
Iron (mg)	4.8 ± 0.2	4.1 ± 0.4	3.3 ± 0.1	7.8 ± 0.1
Zinc (mg)	1.3 ± 0.1	1.5 ± 0.0	1.7 ± 0.1	3.8 ± 0.1
Potassium (mg)	4.0 ± 0.0	0.9 ± 0.0	2.5 ± 0.0	1.0 ± 0.0
Sodium (mg)	0.2 ± 0.0	21 ± 0	11 ± 0	0.0 ± 0.0
Vitamin A (µg RAE) ^2^	210 ± 11	NA ^3^	138 ± 1	NA
Lycopene (mg)	102 ± 9	NA	NA	NA
Lutein (mg)	1.1 ± 0.0	NA	4.3 ± 0.1	NA
Thiamine (mg)	0.2 ± 0.0	0.1 ± 0.0	0.1 ± 0.0	0.3 ± 0.0
Riboflavin (mg)	0.5 ± 0.0	0.1 ± 0.0	0.4 ± 0.0	0.2 ± 0.0
Niacin (mg)	12 ± 1	1.0 ± 0.0	4.0 ± 0.2	2.1 ± 0.1
Pantothenic acid (mg)	0.6 ± 0.0	0.6 ± 0.1	1.5 ± 0.0	0.9 ± 0.0
Pyridoxine (mg)	0.6 ± 0.1	0.5 ± 0.0	0.7 ± 0.0	0.3 ± 0.0
Folic acid (µg)	268 ± 41	152 ± 18	277 ± 23	109 ± 11
Vitamin C (mg)	151 ± 11	<LOQ ^4^	<LOQ	<LOQ
Vitamin K1 (µg)	26 ± 1	NA	NA	NA
Nutrient density score ^5^	315	142	335	394

^1^ Values are means ± SD of two varieties of each dried vegetable type (e.g., the nutritional composition of tomato powder was based on the mean of the values of two tomato powder varieties). ^2^ Vitamin A was calculated from analyzed β-carotene values that were converted to retinol activity equivalents. ^3^ NA = not analyzed. ^4^ LOQ = limit of quantification; for vitamin C this was set at 10 mg/100 g dry vegetables. ^5^ Based on total dietary fiber, magnesium, iron, zinc, potassium, vitamin A, thiamin, riboflavin, niacin, pantothenic acid, pyridoxine, vitamin C, and vitamin K1 as positive nutrients and sodium as a negative nutrient, with the exception that vitamin C and K1 were not included in the score for onion, pumpkin, and lentil; vitamin A was not included for onion and lentil powders.

**Table 2:** Nutrient density scores for dry vegetable powders as compared to respective cooked and fresh vegetables.

	Mean and Ranges of Nutrient Density Scores ^1,2^		
	Dried (Measured *n = 2*)	Cooked (BLS/USDA *n = 2*)	Fresh (BLS/USDA *n = 2*)
Tomato	315 (309–321)	425 (319–531)	473 (408–537)
Onion	142 (130–154)	127 (57–197)	150 (77–223)
Pumpkin	335 (317–353)	320 (336–380)	361 (304–341)
Lentil	394 (365–424)	256 (142–317)	NA ^3^

^1^ The ranges show the mean and lowest and highest nutrient density score for the two dry vegetable powders and for the values in the BLS and USDA for either fresh and cooked vegetables (without fat). ^2^ Values are based on total dietary fiber, magnesium, iron, zinc, potassium, vitamin A, thiamin, riboflavin, niacin, pyridoxine, vitamin C, and vitamin K1 as positive nutrients and sodium as a negative nutrient, with the exception that vitamin C and K1 were not included in the score for onion, pumpkin, and lentil; vitamin A was not included for onion and lentil powders. ^3^ NA, not applicable.

**Table 3:** Mean nutrient content and nutrient density scores of dry vegetable soups.

	Nutrient Content of Dry Vegetable Soups per 250 mL Serving									
	Tomato		Onion		Pumpkin		Pulses/Legumes		Mixed Vegetables	
	Mean ± SD ^1^	% DRI ^2^	Mean ± SD	% DRI	Mean ± SD	% DRI	Mean ± SD	% DRI	Mean ± SD	% DRI
Energy (kcal)	110		58		98		155		90	
Total dietary fiber (g)	2.9 ± 0.2	12	5.2 ± 0.1	21	2.8 ± 0.8	11	11 ± 1	45	4.3 ± 0.5	17
Magnesium (mg)	22 ± 1	6	10 ± 1	3	14 ± 4	4	65 ± 1	17	20 ± 4	5
Iron (mg)	0.70 ± 0.04	5	0.45 ± 0.13	3	0.37 ± 0.03	3	3.17 ± 0.91	23	0.83 ± 0.24	6
Zinc (mg)	0.34 ± 0.13	3	0.25 ± 0.11	3	0.23 ± 0.02	2	1.70 ± 0.58	17	0.29 ± 0.05	3
Potassium (mg)	757 ± 118	22	294 ± 138	8	291 ± 107	8	783 ± 25	22	327 ± 26	9
Sodium (mg)	856 ± 26	43	843 ± 152	42	891 ± 177	45	825 ± 205	41	713 ± 36	36
Vitamin A (µg RAE) ^3^	31 ± 3	15	NA ^4^		22 ± 3	11	NA		28 ± 5	14
Lycopene (mg)	11.3 ± 0.8		NA		NA		NA		NA	
Lutein (mg)	0.14 ± 0.01		NA		0.46 ± 0.00		NA		NA	
Thiamine (mg)	0.07 ± 0.03	7	0.13 ± 0.06	12	0.04 ± 0.02	3	0.19 ± 0.02	17	0.05 ± 0.03	5
Riboflavin (mg)	0.13 ± 0.02	9	0.07 ± 0.05	5	0.07 ± 0.00	5	0.18 ± 0.08	13	0.07 ± 0.01	5
Niacin (mg)	1.47 ± 0.07	9	0.46 ± 0.30	3	0.70 ± 0.13	4	1.65 ± 0.70	10	0.89 ± 0.21	6
Pantothenic acid (mg)	0.18 ± 0.05	3	0.14 ± 0.05	2	0.21 ± 0.03	3	0.46 ± 0.18	8	0.21 ± 0.03	3
Pyridoxine (mg)	0.09 ± 0.00	6	0.07 ± 0.01	5	0.09 ± 0.02	6	0.18 ± 0.04	13	0.12 ± 0.04	8
Vitamin C (mg)	15 ± 1	18	<LOQ ^5^		<LOQ		<LOQ		<LOQ	
Vitamin K1 (µg)	4.7 ± 0.1	6	1.3 ± 0.8	2	NA		11.2 ± 5.0	15	10.5 ± 2.2	14
Nutrient density score ^6^	36		34		9		88		36	

^1^ Values are means ± SD of triplicate measurements of two varieties of each vegetable soup type. ^2^ DRI = dietary reference intake [16]. ^3^ Vitamin A was calculated from analyzed β-Carotene values that were converted to retinol activity equivalents. ^4^ NA = not analyzed. Based on the raw ingredients for these soup types, it was not expected to detect any carotenoids. ^5^ LOQ = limit of quantification. For vitamin C, this was set at 10 mg/100 g dry soup. ^6^ Based on total dietary fiber, magnesium, iron, zinc, potassium, vitamin A, thiamin, riboflavin, niacin, pantothenic acid, pyridoxine, vitamin C, and vitamin K1 as positive nutrients and sodium as a negative nutrient, with the exception that vitamin C was not included in the score for onion, pumpkin, pulses/legumes, and mixed vegetables; vitamin K1 was not included for pumpkin, and vitamin A was not included for onion and pulses/legumes soups.

**Table 4:** Vegetable content of dry vegetable soups per 250 mL serving.

Soup Variety	Dry Vegetable Content (g)	Weighted Factor to Account for Rehydration ^2^	Mean Fresh Vegetable Equivalents (g)	Contribution to Vegetable Recommendation (%) ^3^
Tomato	11.9 ^1^	15.9	189	79
Onion	8.9	8.6	77	32
Pumpkin	9.7	10.7	103	43
Pulses/legumes	40.4	3.7	148	61
Mixed vegetable	9.1	9.2	84	35

^1^ Values are means of two sub varieties of each vegetable soup variety. ^2^ Factor is a weighted mean of factors to account of rehydration of the different dried vegetable powders. ^3^ Based on WHO/FAO of ≥240 g/day [10].

## Supplementary Materials

The following are available online at https://www.mdpi.com/2072-6643/11/6/1270/s1, Data S1: Trade names and ingredient composition of soups used in this study, Data S2: Study of retention of selected marker nutrients in dried and home-made soups, Figure S1: Nutrient retention from fresh vegetable ingredients to prepared dry and home-made soups.
