# Supplementary material for: Nutritional Quality of Dry Vegetable Soups
Source: Nutrients. 2019 Jun 4;11(6):1270. doi: 10.3390/nu11061270 (PMC6627832; doi:10.3390/nu11061270)
Supplement: Supplementary file 1 [file nutrients-11-01270-s001.pdf]

Supplementary material

S1. Trade names and ingredient composition of soups

Tomato soup 1 - Knorr Velours de tomates à la mozzarella (191 g fresh vegetable equivalent per serving):

Vegetable powders used for calculation:
Tomato 33% (moisture 4%)
Onion >1% (moisture 6%)
Leek <1% (moisture 6%)

Other ingredients, not in vegetable calculation: Sugar, wheat flour, vegetable fat, mozzarella, potato starch, yeast extract, salt, processed cheese (cheese, whey), natural mineral salt, lactose, maltodextrin, fructose, cheese, milk proteins, concentrated vegetable juice (celery, carrot, leek, onion), herbs (thyme, oregano), lemon juice, flavors.

Tomato soup 2 - Knorr Feinschmecker Tomatensuppe Toscana (182 g fresh vegetable equivalent per serving):

Vegetable powders used for calculation:
Tomato 39% (moisture 4%)
Onion 2% (moisture 6%)
Beet root <1% (moisture 6%), Celery <1% (moisture 6%)

Other ingredients, not in vegetable calculation: Palm oil, starch, sugar, wheat flour, iodized table salt, glucose syrup, roasted onion, flavors (wheat, barley), milk protein, salt substitutes, seasoning, beetroot powder, herbs (basil, oregano, thyme), salt, garlic, parsley root, yeast extract.

Onion soup 1 - Knorr Feinschmecker Zwiebel Suppe (78 g fresh vegetable equivalent per serving):

Vegetable powders used for calculation:
Onion 44% (moisture 6%)

Other ingredients, not in vegetable calculation: Potato starch, palm oil, iodized salt, yeast extract, wheat flour, flavors, fructose, salt, vegetable juice concentrates (celery, carrots, leek, onions), spices (garlic, pepper, turmeric, cumin), white wine extract, caramel sugar syrup, citric acid, maltodextrin, sunflower oil.

Onion soup 2 - Knorr Rozkosze podniebienia Francuska zupa cebulowa z prażoną cebulką (70 g fresh vegetable equivalent per serving):

Vegetable powders used for calculation:
Onion 49% (moisture 6%)
Carrot 1% (moisture 6%)

Other ingredients, not in vegetable calculation: Starch, salt, wheat flour, palm fat, yeast extract, flavor enhancers (monosodium glutamate, disodium inosinate, disodium guanylate), aroma, garlic, pepper, cumin, coriander, parsley root, sunflower oil, parsley, citric acid, concentrated onion juice, glucose.

Legumes/pulses (lentil) soup 1 - Knorr Großmutters Geheimnis - Deftiger Linsentopf mit Speck (186 g fresh vegetable equivalent per serving):

Vegetable powders used for calculation:
Lentil 70% (moisture 14%)
Onion 3% (moisture 6%)
Carrot 2% (moisture 6%)
Tomato 2% (moisture 4%)
Leek 2% (moisture 6%)

Other ingredients, not in vegetable calculation: Smoked bacon (pork, salt, spice extract, beech smoke), potatoes, flavors,, smoked bacon (pork, salt, smoke), yeast extract, iodized food salt, food salt, smoked bacon fat (fat, smoke), starch, acidifier citric acid, acid controller sodium diacetate, milk sugar, pepper.

Legumes/pulses (bean) soup 2 - Knorr Segreti della Nonna - Pasta e Fagioli (118 g fresh vegetable equivalent per serving):

Vegetable powders used for calculation:
Brown bean 32% (moisture 6%)
White bean 14% (moisture 6%)
Tomato 3% (moisture 4%)
Onion 2% (moisture 6%)

Other ingredients, not in vegetable calculation: Durum wheat semolina pasta, wheat flour, salt, extra virgin olive oil, onion, potassium chloride, bouillon, sugar, yeast extract, parsley, rosemary, concentrated vegetable juices (celery, carrot, onion, leek), potato starch, spices (celery seed, parsley root), flavors.

Pumpkin soup 1 - Knorr Feinschmecker Kürbiscremesuppe (64 g fresh vegetable equivalent per serving):

Vegetable powders used for calculation:
Pumpkin 22% (moisture 6%)

Other ingredients, not in vegetable calculation: Palm oil, potato starch, wheat flour, sugar, iodized salt, glucose syrup, yeast extract, milk protein, salt, spices (garlic, pepper, cumin), maltodextrin, parsley, fructose, soy sauce (soy, wheat), lemon juice powder, flavors.

Pumpkin soup 2 - Knorr Crème de Potiron (143 g fresh vegetable equivalent per serving):

Vegetable powders used for calculation:
Pumpkin 52% (moisture 6%)
Onion 2% (moisture 6%)

Other ingredients, not in vegetable calculation: Potato, vegetable fat, potato starch, salt, sugar, lactose, yeast extract, milk proteins, flavorings (including wheat), vegetable extracts (carrot, celery, onion, leek), nutmeg.

Mixed vegetable soup 1 - Knorr Soupe passée aux 9 légumes (88 g fresh vegetable equivalent per serving)

Vegetable powders used for calculation:
White bean 10% (moisture 6%)
Carrot 10% (moisture 6%)
Onion 8% (moisture 6%)
Celery 5% (moisture 6%)
Leek 4% (moisture 6%)
Tomato 3% (moisture 4%)
Brown bean 2% (moisture 6%)
Green bean < 1% (moisture 6%), turnip < 1% (moisture 6%)

Other ingredients, not in vegetable calculation: Potato, salt, vegetable fat, pork fat, yeast extract, lactose, potato starch, flavors (including wheat), milk proteins, turmeric.

Mixed vegetable soup 2 - Knorr Soupe crème de legumes (79 g fresh vegetable equivalent per serving):

Vegetable powders used for calculation:
Carrot 6% (moisture 6%)
Pumpkin 6% (moisture 6%)

Tomato 5% (moisture 4%)
Brown bean 5% (moisture 6%)
Onion 3% (moisture 6%)
Celery 1% (moisture 6%)
Broccoli 1% (moisture 6%)
Leek <1% (moisture 6%), green bean < 1% (moisture 6%),

Other ingredients, not in vegetable calculation: Potato, palm fat, wheat flour, salt, sugar, glucose syrup, hydrolyzed vegetable protein, potassium chloride, milk protein, potato starch, flavors (including celery), yeast extract, turmeric.

S2. Study of retention of selected marker nutrients in dried and home-made soups

An additional study was conducted to assess the retention of a few key nutrients during the entire process of pre-processing, drying, and cooking of the dried soups compared to nutrient retention during the process of making soups directly from fresh vegetables as with home-made soups. For this study, three soup varieties were selected: Tomato, onion, and lentil. A few micronutrients were selected as key markers of nutrient retention based on previous studies, including vitamin C for tomato and onion soups and folate for lentil soups. Additionally, lycopene in tomato and flavonols in onions were analyzed. Potassium is not expected to degrade and was used as a positive control for nutrient retention in all soups.

The retention of the selected key markers was determined at each step of the drying and cooking process from fresh vegetable to the final cooked dry soup and compared to a home cooked analogue. The cooking procedures of home-made soups were selected with the help of experienced chefs. It was ensured that the home-made soups always contained the same amount of vegetables as its dried equivalents. Vegetables for the dried soups were obtained from the following commercial suppliers: Tomatoes from Agraz, Badajoz, Spain; onions from Mezöker Kft., Hungary; dried lentils from Spokane Seeds, Spokane Valley, WA, USA; and precooked lentils from Walker Seeds Ltd. Tisdale, Canada. The home-made soups were prepared with locally sourced supermarket vegetables. Homogenized samples were taken and analyzed in duplicate, except the flavonols (quadruplicate) at the Unilever Heilbronn Laboratory in Germany. Total vitamin C was determined by extracting the samples with meta-phosphoric acid solution followed by homocysteine treatment and quantification by HPLC-DAD. Total folate and flavonols were measured by HPLC-DAD-MS. Potassium was determined via microwave digestion followed by analysis by ICP-OES, lycopene by HPLC-DAD.

Figure S1 shows the percentage retention from the nutrient levels in the fresh vegetables. In all soups, nutrient levels decreased as compared to the fresh vegetables, except for the positive control, potassium, which remained at 100% throughout the experiment. In tomato and lentil soups, the nutrients were better retained with the dried soup format, while, in onion soup, the nutrients were better retained in the home cooked soups.

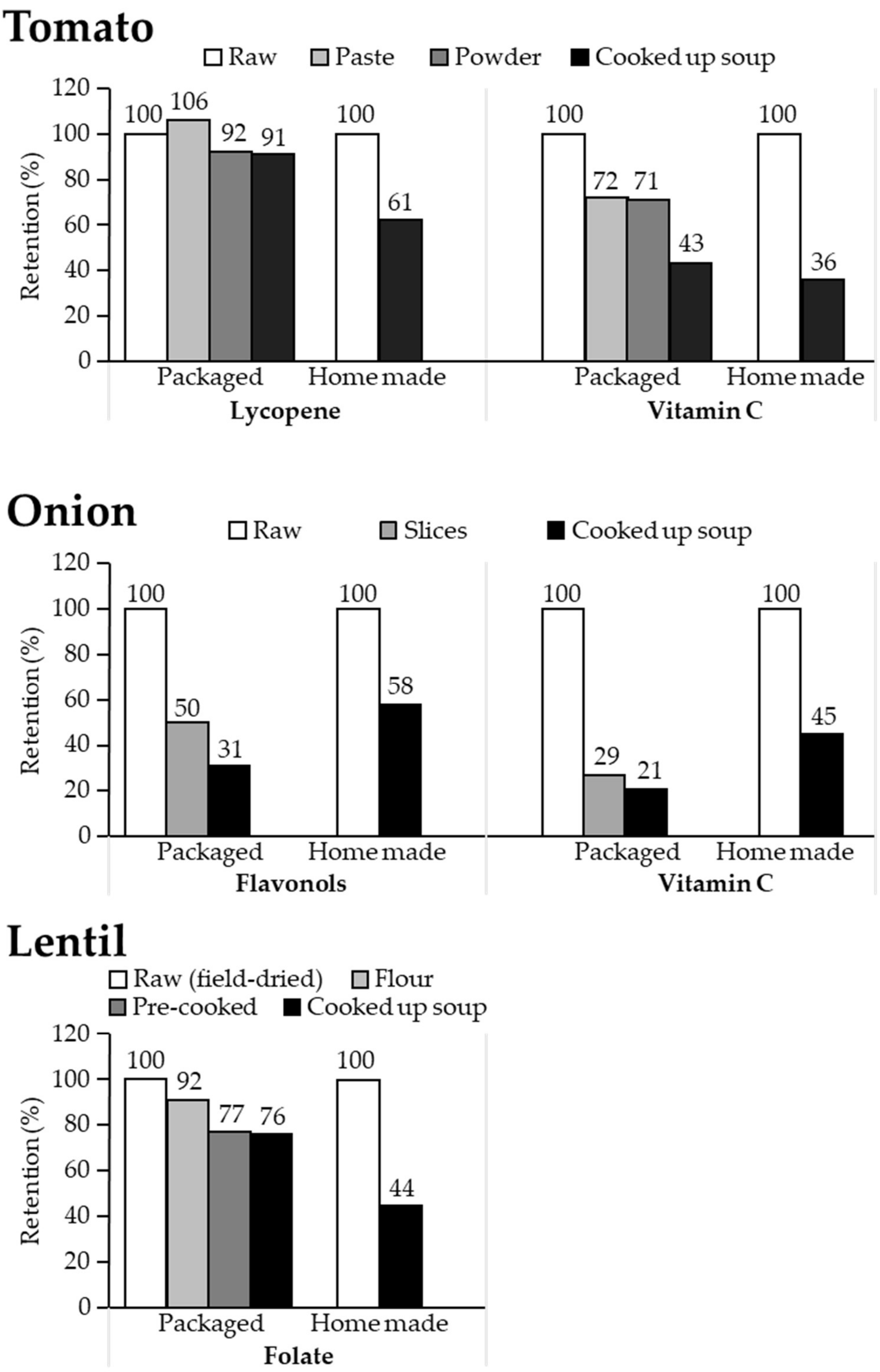

Figure 1. Nutrient retention from fresh vegetable ingredients to prepared dry and home-made soups.
